# Mother-to-child transmission of HIV and its predictors among HIV-exposed infants at a PMTCT clinic in northwest Ethiopia

**DOI:** 10.1186/1471-2458-13-398

**Published:** 2013-04-27

**Authors:** Digsu Negese Koye, Berihun Megabiaw Zeleke

**Affiliations:** 1 Department of Epidemiology and Biostatistics, College of Medicine and Health Sciences, University of Gondar, Gondar, Ethiopia

**Keywords:** HIV-exposed infants, HIV transmission, Predictors, Ethiopia

## Abstract

**Background:**

Mother-to-child transmission of HIV (MTCT) accounts for more than 90% of pediatric Acquired Immunodeficiency Syndrome (AIDS) cases. Prevention of mother to child transmission (PMTCT) programs are provided for dual benefits i.e. prevention of HIV transmission from mother to child and enrolment of infected pregnant women and their families into antiretroviral treatment (ART). This study assessed risk and predictors of HIV transmission among HIV-exposed infants on follow up at a PMTCT clinic in a referral hospital.

**Methods:**

Institution based retrospective follow up study was carried at Gondar University referral hospital PMTCT clinic. All eligible records of HIV-exposed infants enrolled between September 2005 and July 2011 were included. A midwife nurse collected data using a structured data extraction format. Data were then entered in to EPI INFO Version 3.5.1 statistical software and analyzed by SPSS version 20.0. Both bivariate and multivariate analyses were carried out to identify associations.

**Results:**

A total of 509 infant records were included in the analysis. The median age of infants at enrolment to follow up was 6 weeks (inter quartile range [IQR] = 2 weeks). A total of 51(10%, 95% CI: 7.8% - 13%) infants were infected with HIV. Late enrolment to the exposed infant follow up clinic (Adjusted Odds Ratio [AOR] = 2.89, 95% CI: 1.35, 6.21), rural residence (AOR = 5.05, 95% CI: 2.34, 10.9), home delivery (AOR = 2.82, 95% CI: 1.2, 6.64), absence of maternal PMTCT interventions (AOR = 5.02, 95% CI: 2.43, 10.4) and mixed infant feeding practices (AOR = 4.18, 95% CI: 1.59, 10.99) were significantly and independently associated with maternal to child transmission of HIV in this study.

**Conclusions:**

There is a high risk of MTCT of HIV among exposed infants on follow up at the PMTCT clinic of the University of Gondar referral hospital. The findings of this study will provide valuable information for policy makers to enhance commitment and support for rural settings in the PMTCT scaling-up program.

## Background

The HIV pandemic created an enormous challenge to the survival of humankind worldwide. At the end of 2011, an estimated 34 million people were living with HIV globally. There was 2.5 million new HIV infections, including 330, 000 among children less than 15 years. The annual number of people dying from AIDS-related causes worldwide is steadily decreasing. In 2011, 1.7 million people worldwide died from AIDS related causes, down 24% from the peak in 2005 [1].

Besides the dominant heterosexual transmission, vertical HIV transmission from mother to child accounts for more than 90% of paediatric AIDS. PMTCT programs provide for both prevention of HIV transmission from mother to child and enrolment of infected pregnant women and their families into antiretroviral treatment (ART). It is undertaken by the government of Ethiopia in an effort to mitigate the impacts of the epidemic in the general population and amongst children in particular. The availability and use of short-course antiretroviral (ARV) prophylaxis-a safe and well-tolerated regimen-can contribute significantly to PMTCT during childbirth [2].

Ethiopia is one of the countries most severely hit by the epidemic. According to Ethiopian demographic and health survey 2011, the national adult HIV prevalence is reported to be 1.5% (4.2% in urban and 0.6% in rural areas) [3]. Highest prevalence occurs in the 15–24 age groups and prevalence is higher among females than males in both urban and rural areas. Prevalence appears to have levelled off in urban areas but continues to rise in rural areas, where 85% of the population lives [2].

Ethiopia has adopted the World Health Organization (WHO) four pronged PMTCT strategy as a key entry point to HIV care for women, men and families. These are: primary prevention of HIV infection, prevention of unintended pregnancies among HIV-infected women, prevention of HIV transmission from HIV-infected women to their offspring, provision of care and support to women infected with HIV, their infants and families [4].

Although there are reports on the effectiveness and outcomes of PMTCT from many countries, there are limited studies conducted in Ethiopia to assess the risk of HIV transmission and its predictors among exposed infants. One retrospective study conducted in Addis Ababa, Ethiopia, showed 11.8% HIV prevalence among exposed infants [5]. Provision of baseline information about the risk and predictors of HIV transmission among HIV exposed infants is of immense importance. This study thus aimed to assess the risk of HIV transmission and its predictors among HIV exposed infants who had follow up at PMTCT clinic of Gondar University Hospital.

## Methods

Institution based retrospective follow up study was carried out among infants and mothers who were on follow up at the PMTCT clinic.

### Study area and setting

The study was conducted at Gondar University referral hospital PMTCT clinic from March to April 2012. Gondar, situated at 748 km from Addis Ababa, (the Ethiopian capital) has about a total population of 207, 044 (in 2007). The hospital is located in Gondar city, Amhara National Regional State, northwest Ethiopia. It is one of oldest health institutions in northwest Ethiopia, which is the referral centre for four district hospitals in the area providing service for over 5 million people in the catchment area. Apart from other services, the hospital is providing HIV chronic care including PMTCT services for clients since 2005 as one component of comprehensive HIV/AIDS care and support program.

### Study population

All records of HIV-exposed infants enrolled between September 2005 and July 2011 at the PMTCT clinic of Gondar University referral hospital were included in this study. The records of infants transferred in from other sites were excluded.

### Follow up of HIV-positive mothers and their infants in northwest Ethiopia

PMTCT protocols were based on WHO guidelines evolved over the duration of the study period in response to changing international guidelines and newly available information. Throughout the course of the program, women who were eligible for HIV treatment based on their own clinical and immunologic status received combination ART.

According to the guidelines, follow up schedule of infants born to HIV-positive mothers is: at 6 hours after birth, 6^th^ day and then at the 6^th^, 10^th^ and 14^th^ week of life. Thereafter, it is on monthly basis until 6^th^ month of age and every 3 months until age of 18 months for asymptomatic infants. However, children were followed for variable periods of time as their HIV infection status may be declared at different times. Cotrimoxazole prophylaxis is usually started when the infant is aged 4–6 weeks. HIV testing of infants born to HIV-positive mothers was according to the guidelines for PMTCT of HIV in Ethiopia [2]. Deoxyribonucleic acid (DNA) Polymerase Chain Reaction (PCR) testing is done at 6 weeks or as early as possible thereafter. The child will be referred for HIV/ART care clinic if child has a positive virological test or suspected of having symptomatic HIV or displays any severe classifications possibly due to HIV or has positive antibody test under 18 months and 2 or more of the following: oral thrush, severe pneumonia or severe sepsis.

### Operational definitions

#### ARV prophylaxis

Short term use of ARV drugs in the mother and/or infant to reduce MTCT [2].

### Data collection and management

Data on socio-demographic information, birth weight of the infant, infant prophylaxis, maternal PMTCT interventions, HIV test result and related interventions were collected using a structured data collection format. The format was pretested at Gondar health center on twenty records and improved accordingly. Data were collected by a midwife nurse who works at the PMTCT clinic of the hospital. Data were then checked, coded and entered in to EPI info version 3.5.1 version and analyzed by SPSS version 20.0. Most of the variables were fitted to the bivariate logistic regression. Then all variables having p value ≤ 0.2 in the bivariate analysis were further entered into multivariate logistic regression model. Backward Logistic Regression (LR) method was employed and variables having p value < 0.05 were taken as significant predictors. Crude and adjusted odds ratios with their 95% confidence intervals were calculated.

### Ethical considerations

Ethical clearance was obtained from Institutional Review Board of the University of Gondar. Permission letter was obtained from the hospital administration. As this was a retrospective study using secondary data, informed consent from individual patients was not possible. Names and medical registration numbers of patients were not included in the study.

## Results

A total of 521 mother-infant pairs were enrolled in to the PMTCT clinic during the study period. Among these, 509 records were included in the final analysis. Twelve records were incomplete and excluded. The median age at enrolment to HIV-exposed infant follow up clinic was 6 weeks (IQR = 2 weeks). Nearly half (51.7%) of them were males and majority (83.3%) were from urban areas. Three hundred sixty eight (81.2%) of the mothers were enrolled to ART clinic. However, 92(18.1%) mothers did not receive any PMTCT interventions during pregnancy and child birth. Majority (90.4%) of the exposed infants were born at a health institution. Almost all (97.8%) mothers were alive when infants started follow up and majority (85.1%) of the infants took ARV prophylaxis at birth (Table 1).

**Table 1:** Socio-demographic and clinical characteristics of HIV exposed infants and their mothers who had a follow up at Gondar University Referral Hospital

**Variables**	**Frequency**	**Percent**
**Age at enrolment**		
≤ 6 weeks	353	69.4
> 6 weeks	156	30.6
**Sex**		
Male	263	51.7
Female	246	48.3
**Residence**		
Urban	424	83.3
Rural	85	16.7
**Place of Birth**		
Hospital	403	79.2
Health center	57	11.2
Home	49	9.6
**Maternal status**		
Alive	498	97.8
Dead	11	2.2
**Maternal enrolment to ART care (n = 453)**		
Enrolled	368	81.2
Not enrolled	85	18.8
Maternal PMTCT intervention*		
None	92	18.1
sdNVP	85	16.7
sdNVP + AZT for 7 days	10	2.0
sdNVP + AZT for 4 weeks	3	.6
Already on ART	189	37.1
sdNVP + AZT for 7 days + 3TC	130	25.5
**Infant ARV prophylaxis at birth (n = 495)**		
Received	421	85.1
Not Received	72	14.5
Not known	2	0.4
Infant ARV prophylaxis during follow up (n = 491)*		
None	75	15.3
sdNVP	99	20.2
sdNVP + AZT for 7 days	277	56.4
sdNVP + AZT for 4 weeks	40	8.1

*AZT, Zidovidine; *sdNVP*, Single dose Neverapine; *3TC*, Lamuvidine.

At enrollment, most (78.8%) infants were on exclusive breast feeding, 67(13.3%) on exclusive replacement feeding, while the rest 40(7.9%) were on mixed feeding. Forty three (8.4%) infants had diagnosis suggestive of HIV infection. The most common diagnosis was Pneumonia/Lower respiratory tract infections, followed by persistent diarrhea and hepato-spleenomegaly. More than three quarters (80.5%) of the infants started cotrimoxazole prophylaxis at enrolment.

### Risk of maternal to child HIV transmission

Of all 509 infants, 51(10%, 95% CI: 7.8% - 13%) were HIV-infected. Most were diagnosed by both DNA/PCR at their six week of age and rapid antibody test at a later age. Among those HIV positive after PMTCT follow up, most were from rural than urban (28.2% Vs 6.4%) residence.

### Predictors of maternal to child HIV transmission

As clearly depicted on the multivariate logistic regression, late enrolment to the exposed infant follow up clinic, rural residence, delivery at home, absence of maternal PMTCT intervention and mixed infant feeding practices were significantly and independently associated with maternal to child HIV transmission. However, sex, maternal status (living or dead) and infant baseline cotrimoxazole prophylaxis were not significantly associated with MTCT.

Accordingly, infants who started follow up after six weeks of age were at a 2.89 (AOR = 2.89, 95% CI: 1.35, 6.21) times higher risk of MTCT as compared to their counterparts. Infants of rural dwelling mothers were at a 5.05 (AOR = 5.05, 95% CI: 2.34, 10.9) times higher risk of vertical HIV transmission as compared to urban dwelling mothers. Infants delivered at home had 2.82 (AOR = 2.82, 95% CI: 1.2, 6.64) times higher risk of MTCT compared to those delivered at health institution. If the mother did not receive any form of PMTCT intervention during pregnancy or child birth there is a fivefold (AOR = 5.02, 95% CI: 2.43, 10.4) risk of MTCT as compared to at least one form of PMTCT intervention. Mixed feeding was also another important predictor in which the risk of MTCT was about four (AOR = 4.18, 95% CI: 1.59, 10.99) times higher as compared to exclusive breastfed (Table 2).

**Table 2:** Multivariate logistic regression analysis of predictors of vertical transmission at Gondar University Referral Hospital, Northwest Ethiopia

**Variables**	**HIV status**		**Crude OR (95% CI)**	**Adjusted OR (95% CI)**
	**Positive**	**Negative**		
**Age at enrolment**				
≤ 6 weeks	15	338	1.0	1.0
> 6 weeks	36	120	6.76 (3.57, 12.79)*	2.89 (1.35, 6.21)*
**Sex**				
Male	26	237	0.97 (0.54, 1.73)	**
Female	25	221	1.0	
**Residence**				
Urban	27	397	1.0	1.0
Rural	24	61	5.79 (3.14, 10.67)*	5.05 (2.34, 10.9)*
**Place of Birth**				
Health institution	33	427	1.0	1.0
Home	18	31	7.5 (3.81, 14.83)	2.82 (1.2, 6.64)
**Maternal status**				
Alive	47	451	1.0	**
Dead	4	7	5.48 (1.55, 19.42)*	
**Maternal PMTCT intervention**				
Yes	20	397	1.0	1.0
No	31	61	10.09 (5.41, 18.82)*	5.02 (2.43, 10.4)*
**Infant feeding practice**				
Exclusive breast feeding	26	371	1.0	1.0
Exclusive replacement feeding	8	59	1.94 (0.84,4.48)	1.72 (0.65, 4.53)
Mixed feeding	16	24	9.51 (4.51,20.08)*	4.18 (1.59, 10.99)*
**History of infant ARV prophylaxis at birth**				
Received	21	400	1.0	
Not Received	30	44	12.99 (6.86, 24.6)*	**
**Infant ARV prophylaxis during follow up**				
None	30	45	8.22 (2.32, 29.10)*	**
sdNVP	5	94	0.66 (0.15, 2.89)	
sdNVP + AZT 7 days	12	265	0.56 (0.15, 2.07)	
sdNVP + AZT 4 weeks	3	37	1.0	
**Baseline Cotrimoxazole**				**
Yes	44	364	1.0	
No	7	92	0.63 (0.28, 1.44)	

*Significant at P-Value <0.05.

* *non significant from the multivariate logistic regression (Backward LR method).

## Discussion

Prevention of mother-to-child transmission of HIV-1 is a major public health challenge in many resource-poor countries including Ethiopia. This study was based on a retrospective analysis of data from a routine PMTCT program in a referral hospital. According to this study, among HIV-exposed infants on follow up at the PMTCT clinic of Gondar University referral hospital, one-tenth of HIV-exposed infants got HIV-infected. We included only those infants whose mothers were tested HIV-positive and followed up until the infant’s HIV status was confirmed.

Compared to high income countries (<2%) [6,7], and reports from India (6.5%) and South Africa (5.9%) [8,9], in our study risk of MTCT was very high. This may be due to the universal use of highly active antiretroviral therapy for pregnant women, elective caesarean sections and avoidance of breastfeeding in developed countries [10]. However, such preventive approaches are limited in poor countries due to poor funding, social and cultural norms [11].

Factor that put an infant to a higher risk of HIV transmission were; late enrolment to the follow up clinic, rural residence, home delivery, absence of maternal PMTCT interventions and mixed infant feeding practices. The reasons for the high relevance of these factors could be overlapping to one another. Firstly, infants born from rural mothers are prone for mixed feeding. Secondly, rural mothers are less likely to screen and enroll themselves to antenatal care or health institution delivery and thereby less likely to benefit from PMTCT interventions. As a result many women in resource-poor countries do not have access to prenatal care and often present with infection lately [12]. All these health care disadvantages in the rural settings compared to the urban areas would put infants of rural mothers at a higher risk of MTCT of HIV and thereby mortality [10].

Similar to studies done so far in different corners of the world [9,13,14], if the mother received PMTCT interventions during pregnancy, the risk of MTCT of HIV was only 4.8%. The result of this study is in line with the widely accepted fact that providing ARVs to both the mother and the newborn prevents MTCT of HIV [15]. In this study, the risk of MTCT was 40.5% among infants with no prophylaxis, 5.1% for infants who received sdNVP prophylaxis and 4.9% for infants who took combined (sdNVP + AZT) prophylaxis. The difference was significant between no prohylaxis and sdNVP groups. However, there was no statistical difference between sdNVP and combined (sdNVP + AZT) prophylaxis regimens. This is similar to previous studies reporting a higher benefit of ARV prophylaxis to exposed infants [5,9,13] especially with combined regimens. A cohort study in Abidjan, Côte d’Ivoire, reported a 72% reduction of MTCT [16] with ZDV + sdNVP compared to ZDV alone while another study reported an increasing drug number and duration would significantly decrease the risk of MTCT [9]. However, this difference was not observed in the current study partly due to the lower sample size.

### Limitations

As we used secondary data from a PMTCT clinic, it was difficult to control for inconsistencies and missing values. Maternal HIV-1 viral load and duration of disease and child’s vaccine status were not systematically recorded and could not be taken into account. This study did not aim to differentiate when MTCT occurred i.e. pre-partum, intra-partum or post partum period. The fact that all potential factors were not included and assessed may affect generalization of predictors in this study. Despite these limitations, to the best of our knowledge, this study presented primary results of the effectiveness of routine PMTCT interventions in Gondar University referral hospital.

## Conclusion

There is a high risk of MTCT of HIV among exposed infants on follow up at the PMTCT clinic of the University of Gondar referral hospital. The risk of MTCT of HIV was higher among those from rural areas, enrolled after six weeks of age, had no maternal PMTCT intervention, delivered at home and mixed fed infants. The findings of this study will provide valuable information for policy makers in the PMTCT scaling-up program to focus on rural settings.

## Competing interests

The authors declare that they have no competing interests.

## Author’s contributions

Both authors contributed equally in the design, data collection and write up of this manuscript. Both authors read and approved the final manuscript.

## Pre-publication history

The pre-publication history for this paper can be accessed here:

http://www.biomedcentral.com/1471-2458/13/398/prepub
